# Effects on human transcriptome of mutated BRCA1 BRCT domain: A microarray study

**DOI:** 10.1186/1471-2407-12-207

**Published:** 2012-05-30

**Authors:** Caterina Iofrida, Erika Melissari, Veronica Mariotti, Chiara Guglielmi, Lucia Guidugli, Maria Adelaide Caligo, Silvia Pellegrini

**Affiliations:** 1 Department of Experimental Pathology, Medical Biotechnology, Epidemiology and Infectious Diseases, University of Pisa, 56126, Pisa, Italy; 2 Section of Genetic Oncology Division of Surgical, Molecular and Ultrastructural Pathology, Department of Oncology, University of Pisa and Pisa University Hospital, 56126, Pisa, Italy; 3 Laboratory of Medicine and Pathology, Mayo Clinic, Rochester, MN, USA

**Keywords:** Gene expression, Microarray analysis, Missense mutations, BRCA1 gene, DNA damage, DNA repair, Genomic instability, Cell proliferation, Breast neoplasms, Apoptosis

## Abstract

**Background:**

BRCA1 (breast cancer 1, early onset) missense mutations have been detected in familial breast and ovarian cancers, but the role of these variants in cancer predisposition is often difficult to ascertain. In this work, the molecular mechanisms affected in human cells by two BRCA1 missense variants, M1775R and A1789T, both located in the second BRCT (BRCA1 C Terminus) domain, have been investigated. Both these variants were isolated from familial breast cancer patients and the study of their effect on yeast cell transcriptome has previously provided interesting clues to their possible role in the pathogenesis of breast cancer.

**Methods:**

We compared by Human Whole Genome Microarrays the expression profiles of HeLa cells transfected with one or the other variant and HeLa cells transfected with BRCA1 wild-type. Microarray data analysis was performed by three comparisons: M1775R versus wild-type (M1775RvsWT-contrast), A1789T versus wild-type (A1789TvsWT-contrast) and the mutated BRCT domain versus wild-type (MutvsWT-contrast), considering the two variants as a single mutation of BRCT domain.

**Results:**

201 differentially expressed genes were found in M1775RvsWT-contrast, 313 in A1789TvsWT-contrast and 173 in MutvsWT-contrast. Most of these genes mapped in pathways deregulated in cancer, such as cell cycle progression and DNA damage response and repair.

**Conclusions:**

Our results represent the first molecular evidence of the pathogenetic role of M1775R, already proposed by functional studies, and give support to a similar role for A1789T that we first hypothesized based on the yeast cell experiments. This is in line with the very recently suggested role of BRCT domain as the main effector of BRCA1 tumor suppressor activity.

## Background

*BRCA1* is a tumor suppressor gene whose mutations lead to breast and/or ovarian cancer. Human *BRCA1* encodes a full-length protein of 1863 amino acids that is an important player in controlling cell cycle progression. It is involved in DNA damage response signaling network, participating in G1/S, S and G2/M checkpoints. BRCA1 is required for TP53 phosphorylation mediated by ATM/ATR (ataxia telangiectasia mutated and ataxia telangiectasia and Rad3 related) in response to DNA damage by ionizing or ultraviolet irradiation [1]. BRCA1 is also required for the TP53-mediated activation of CDKN1A (cyclin-dependent kinase inhibitor 1A) transcription that leads to cell cycle arrest [2]. Both BRCA1-ATM and BRCA1-ATR interactions produce the phosphorylation of BRCA1 on specific Ser/Thr residues, required for cell cycle arrest in S and G2 [3]. BRCA1 is also involved in maintaining the cell genomic integrity. It forms a complex with RBBP8 (retinoblastoma binding protein 8) and MRN (MRE11A/RAD50/NBN: meiotic recombination 11 homolog A (S. cerevisiae), RAD50 homolog (S. cerevisiae), nibrin) that partecipates in DNA double-strand break repair mediated by homologous recombination [4]. BRCA1 is furthermore able to act as ubiquitin ligase when heterodimerizes with BARD1 (BRCA1 associated RING domain 1) [5]. The most recent hypothesis on BRCA1 concerns a role in maintaining global heterochromatin integrity that might justify its tumor suppressor function [6].

BRCA1 consists of different functional domains: a N-terminal RING finger domain, two nuclear localization signals, a “SQ” cluster, a branched DNA-binding domain and a C-terminal domain containing two BRCT (BRCA1 C Terminus) repeats [7]. BRCT repeats have been found in many other proteins that regulate DNA damage response and have a crucial role for their function [8]. BRCT repeats have been also described as phosphopeptide-interacting motifs, facilitating the assembly of DNA damage signaling complexes following checkpoint kinases activation [9]. BRCT domains are also involved in the transcriptional activity of BRCA1 and the second BRCT repeat (aa 1760–1863) is critical for the activation of the *CDKN1A* promoter [2]. Finally, a recent paper reported that BRCA1 tumor suppression depends on BRCT phosphoprotein binding [10].

Due to the relevance of this region for BRCA1 function, the study of mutations located in the BRCT domain appears of particular interest.

Aim of this work was to investigate the effects on human cell transcriptome of two missense variants, M1775R and A1789T, both located within the second BRCA1 BRCT domain and isolated from familial breast cancers. In a previous work we examined the expression profiles induced by these two mutations in yeast cells [11]. In a recent paper from Guidugli et al. [12] these two variants were tested in a homologous recombination and a non-homologous end-joining assay in Hela cells. The A1789T variant significantly altered the non-homologous end-joining activity as compared to BRCA1 wild-type.

Here, we compared the expression profiles of HeLa cells transfected with one or the other *BRCA1* variant with that of HeLa cells transfected with *BRCA1* wild-type. We found alterations of molecular mechanisms critical for cell proliferation control and genome integrity, suggestive of a putative role of these two variants in breast cancer pathogenesis.

## Methods

### BRCA1 missense variants

Both *BRCA1* variants are located within the second BRCT domain and, while M1775R has been widely described as deleterious [13], A1789T has been studied only by our group. In yeast cells both these mutations reverted the growth suppression (small colony) phenotype, but only M1775R induced homologous recombination [14]. In HeLa cells A1789T significantly altered the non-homologous end-joining activity as compared to BRCA1 wild-type [12].

### HeLa cells transfection

Five aliquots of the same clone of HeLa G1 cells were transiently transfected with the pcDNA3-BRCA1 wild-type (wt) vector, five with the pcDNA3-BRCA1-M1775R derivative vector and five with the pcDNA3-BRCA1-A1789T derivative vector as described by Guidugli et al. [12].

Twenty-four hours after transfection, cells were washed twice in PBS 1X, pelleted and immediately used to extract RNA or proteins. The increased expression of BRCA1 was assessed by Western Blot as showed by Guidugli et al. [12].

### Microarray

Gene expression was investigated by Whole Human Genome Oligo Microarrays G4112F (Agilent Technologies, Palo Alto, CA, USA). A reference design was adopted using as reference a pool of all the RNA samples from wild-type clones.

Total RNA was extracted and DNase purified with PerfectPure RNA Cultured Cell Kit (5 PRIME) (Eppendorf, Hamburg, Germany). All RNAs, measured by NanoDrop ND-1000 Spectrophotometer (NanoDrop Technologies, Inc. Wilmington, Del, USA), displayed a 260/280 OD ratio > 1.9. The RNA integrity was verified by 1.2% agarose-formaldehyde gel electrophoresis.

Total RNA samples were amplified and labelled with Quick-Amp Labeling kit (Agilent Technologies, Palo Alto, CA, USA). One hundred μl of In Situ Hybridisation Kit Plus mix (Agilent Technologies, Palo Alto, CA, USA) containing 825 ng of Cy3-labelled aRNA (ranging from 11 to 14 Cy3 pmoles) and 825 ng of Cy5-labelled aRNA (18 Cy5 pmoles) were hybridized to each array at 65 °C for 17 h under constant rotation. The arrays were then washed 1 min at RT in 6X SSPE, 0.005% TritonX-102; 1 min at 37 °C in 0.06X SSPE, 0.005% Triton X-102; 30 sec at RT in Acetonitrile solution (Agilent Technologies, Palo Alto, CA, USA) and 30 sec at RT in Stabilization and Drying solution (Agilent Technologies, Palo Alto, CA, USA).

Microarray images were acquired by the Agilent scanner G2565BA and intensity raw data were extracted by the software Feature Extraction V10.5 (Agilent Technologies, Palo Alto, CA, USA). Data preprocessing and statistical analysis were performed by LIMMA (LInear Model of Microarray Analysis) [15] tool. The intensity raw data were background-subtracted and normalized within-arrays and between-arrays.

The contrast matrix was set to evaluate three comparisons: M1775R*vs*WT, A1789T*vs*WT and Mut*vs*WT, considering the two variants as a whole in the latter case. Statistical significance to each gene in each comparison was assigned by B-statistic [16] and only genes with B-statistic > 0 were included.

The pathway analysis was done by Pathway-Express [17,18]. The identification of the Gene Ontology terms that are significantly over- or under-expressed in the lists of differentially expressed genes (DEGs) was performed with Onto-Express using an hypergeometric statistical model [19,20]. The network of biological interactions among DEGs and relevant biological terms was observed by Coremine [21].

### RT-qPCR

RT-qPCR was performed by the iCycler iQ instrument (Biorad, Hercules, CA, USA) and the iQ SYBR Green Supermix (Biorad, Hercules, CA, USA). Total RNAs were reverse transcribed by QuantiTect Reverse Transcription kit (Qiagen, Valencia, CA, USA). PCR primers (listed in Table 1) were designed by Beacon Designer 4.0 (Premier Biosoft International, Palo Alto, CA, USA). RT-qPCR experiments were performed according to MIQE guidelines [22].

**Table 1:** Primer sequences

**Gene Symbol**	**Gene Name**	**Primer Sequences**
Housekeeping genes		
*ACTB*	actin, beta	F: 5'-AACTGGAACGGTGAAGGTGAC-3'
		R: 5'-GACTTCCTGTAACAACGCATCTC-3'
*HPRT1*	hypoxanthine phosphoribosyltransferase 1	F: 5'-ACATCTGGAGTCCTATTGACATCG-3'
		R: 5'-TTAAACAACAATCCGCCCAAAGG-3'
*GAPDH*	glyceraldehyde-3-phosphate dehydrogenase	F: 5'-GTGAAGGTCGGAGTCAACG-3'
		R: 5'-GGTGAAGACGCCAGTGGACTC-3'
*TBP*	TATA box binding protein	F: 5'-GGTGTTGTGAGAAGATGGATGTTG-3'
		R: 5'-CCAGATAGCAGCACGGTATGAG-3'
Target genes		
*CDKN1A*	cyclin-dependent kinase inhibitor 1A (p21, Cip1)	F: 5'-ACTAGGCGGTTGAATGAGAGGTTC-3'
		R: 5'-CAGGTCTGAGTGTCCAGGAAAGG-3'
*EDN1*	endothelin 1	F: 5'-CCAACCATCTTCACTGGCTTCC-3'
		R: 5'-GTCAGACACAAACACTCCCTTAGG-3'
*EEF1E1*	eukaryotic translation elongation factor 1 epsilon 1	F: 5'-TGCGGGAGGTTCTTGTTCTG-3'
		R: 5'-CTGTTAGACTTGGACCATTGTTTG-3'
*GPR56*	G protein-coupled receptor 56	F: 5'-CTACAGCCGAAGAATGTGACTC-3'
		R: 5'-GCAGAAGCAGGATGTTTGGG-3'
*MRE11A*	MRE11 meiotic recombination 11 homolog A (S. cerevisiae)	F: 5'-GATGATGAAGTCCGTGAGGCTATG-3'
		R: 5'-TGTTGGTTGCTGCTGAGATGC-3'
*NFKB1*	nuclear factor of kappa light polypeptide gene enhancer in B-cells 1	F: 5'-CCGTTGGGAATGGTGAGGTC-3'
		R: 5'-TTGAGAATGAAGGTGGATGATTGC-3'
*OBFC2B*	oligonucleotide/oligosaccharide-binding fold containing 2B	F: 5'-GACGATGTTGGCAATCTG-3'
		R: 5'-TGGCTCACTGAAGTTAGG-3'
*PML*	promyelocytic leukemia	F: 5'-CCAAGGCAGTCTCACCAC-3'
		R: 5'-TTCGGCATCTGAGTCTTCC-3'
*SOD2*	superoxide dismutase 2, mitochondrial	F: 5'-GGTGTCCAAGGCTCAGGTTG-3'
		R: 5'-GTGCTCCCACACATCAATCCC-3'

Four housekeeping genes (see Table 1), tested for stability by geNorm [23], were used to normalize the differential expression of target genes. The analysis was performed considering the variants separately for the M1775R*vs*WT- and the A1789T*vs*WT- contrasts, but as a whole for the Mut*vs*WT-contrast. One-tailed Wilcoxon signed rank test was applied to evaluate the statistical significance of results adopting a threshold of 0.05.

### Western blot

Western Blot was performed as previously reported [12].

The level of protein expression was analyzed for: GPR56 (anti-GPR56 rabbit polyclonal antibody H-93: sc-99089, Santa Cruz Biotechnology, Inc., Santa Cruz, CA, USA, dilution 1:1000), MRE11A (anti-MRE11A mouse monoclonal antibody 18: sc-135992, Santa Cruz Biotechnology, Inc., Santa Cruz, CA, USA, dilution 1:500); NFKB1 (anti-NFKB1 mouse monoclonal antibody E-10: sc-8414, Santa Cruz Biotechnology, Inc., Santa Cruz, CA, USA, dilution 1:100) and PML (anti-PML mouse monoclonal IgG2b clone 36.1-104, Upstate Biotechnology, Inc., Waltham, MA, USA, dilution 1: 500).

## Results

### Microarray results

**Figure 1:**
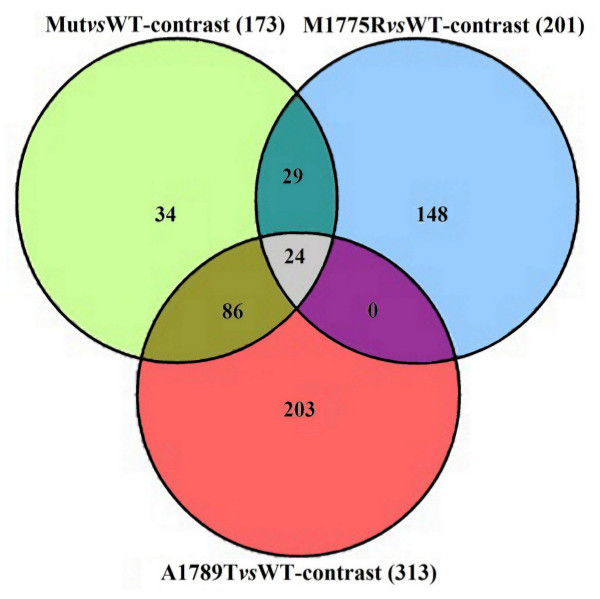
Venn diagram showing the numbers of DEGs shared by the three comparisons.

Mut*vs*WT-contrast showed 173 DEGs (Additional file 1), M1775R*vs*WT-contrast 201 DEGs (Additional file 2) and A1789T*vs*WT-contrast 313 DEGs (Additional file 3). Twenty-four of these genes were differentially expressed with similar fold changes in all the three comparisons (Figure 1) (Additional file 4).

Complete information about the microarray experiments and results can be retrieved from the ArrayExpress database at the European Bioinformatics Institute [24] by the following accession number: E-MTAB-761.

Pathway analysis mapped 27 DEGs in 37 KEGG pathways for Mut*vs*WT (Additional file 1), 40 DEGs in 58 KEGG pathways for M1775R*vs*WT (Additional file 2) and 52 DEGs in 62 KEGG pathways for A1789T*vs*WT (Additional file 3). In all the three comparisons many pathways with high impact factor were involved in cancer.

Twenty-eight pathways were in common among the three comparisons as indicated in FigureFigure 2 (Additional file 5).

**Figure 2:**
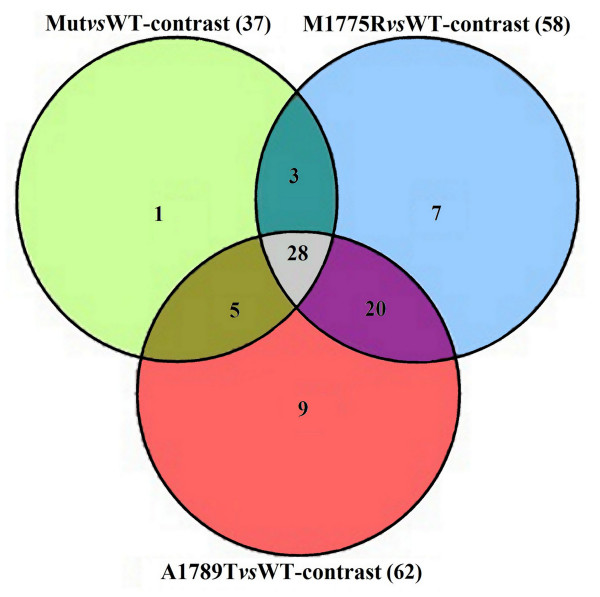
Venn diagram showing the numbers of pathways shared by the three comparisons.

Coremine identified 3594 and 2045 genes linked to biological terms concerning “Cell Proliferation” and “DNA damage and repair” processes, respectively (Additional files 6 and 7). Intersections among these two lists and the three lists of DEGs are shown in Additional files 6 and 7.

### Microarray data validation

**Figure 3:**
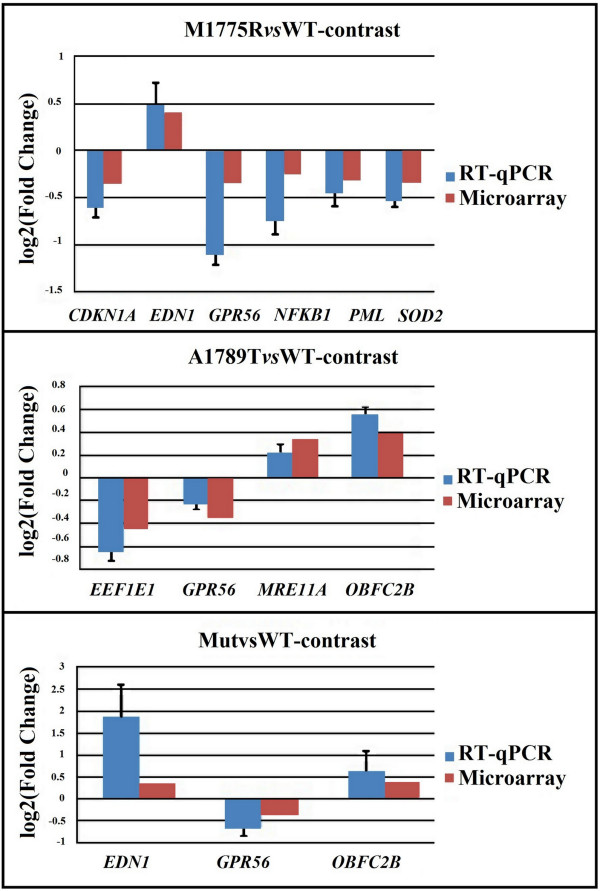
**Microarray and RT-qPCR log2-Fold changes for the nine validated genes.** All the log2-Fold changes are statistically significant (p-value < 0.05).

The differential expression of nine transcripts (Table 1) identified by microarray analysis was validated by RT-qPCR and consistently confirmed for all the thirteen validations (six for M1775R*vs*WT, four for A1789T*vs*WT, three for Mut*vs*WT) (Figure 3).

**Figure 4:**
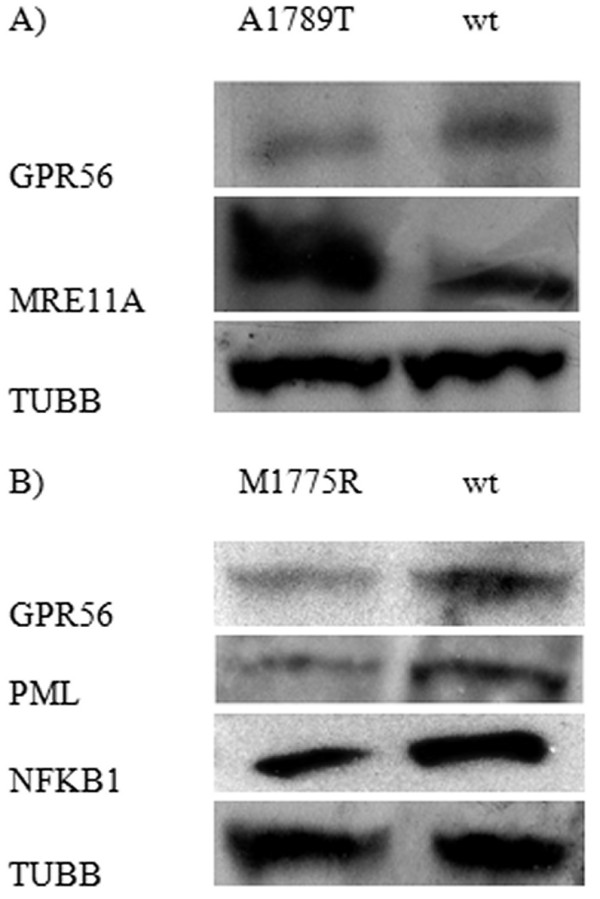
Western Blot analysis of the differential expression of GPR56, MRE11A, PML and NFKB1 proteins.

The differential expression of GPR56, MRE11A, PML and NFKB1 proteins was also confirmed by Western Blot analysis (Figure 4).

## Discussion

Aim of this study was the analysis of the effects on human cell transcriptome of two missense variants located in the second BRCT domain of BRCA1, M1775R and A1789T. Specifically, the gene expression profiles of HeLa cells transfected with one or the other variant were compared with that of HeLa cells transfected with *BRCA1* wild-type. Three different statistical contrasts were performed: M1775R*vs*WT, A1789T*vs*WT and Mut*vs*WT, considering the two variants as a single mutation in the latter case.

**Figure 5:**
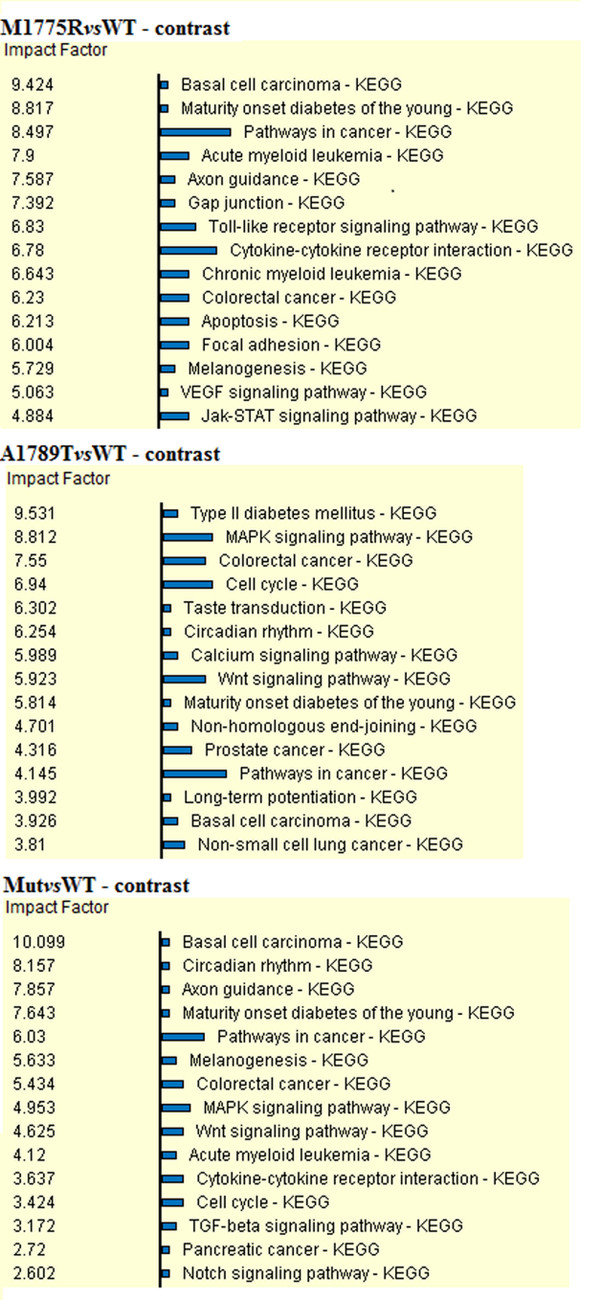
**Diagram showing the top fifteen most impacted pathways for each contrast.** The blue bar is proportional to the number of DEGs mapped in each pathway.

Pathway analysis retrieved many pathways involved in cancer onset and progression as well as linked to specific tumors, as shown in Figure 5.

The information retrieved by pathway analysis was completed by ontological and data-mining analyses, which highlighted three functional categories: cell cycle regulation, apoptosis and DNA damage response and repair, typically deregulated in cancer cells. Cell cycle and apoptosis deregulation leads to aberrant cell proliferation, while an impaired DNA damage response and repair is known to cause genomic instability. All these processes are closely connected, as apoptosis, constituting a defense from anomalous proliferation, is linked to cell cycle block and is activated in response to DNA damage.

### Aberrant cell proliferation

Cancer cells proliferate abnormally. In these cells, the mechanisms ensuring correct cell division, which involve cell cycle arrest at checkpoints, are impaired and there is overexpression of mitogenic factors, such as cell cycle positive regulators. Moreover, in cancer cells apoptosis is often downregulated [25-27].

In our data, a considerable number of differentially expressed genes is strictly linked to cell proliferation.

The DEGs linked to cell proliferation were involved in three main phenomena: cell cycle arrest impairment, cell proliferation enhancement and apoptosis blocking (Table 2).

**Table 2:** Genes linked to aberrant cell proliferation

**Biological Process**	**Gene Symbol**	**Gene Name**	**Contrast**	**log2 (Fold Change)**
Cell cycle arrest impairment	*CDKN1A*	cyclin-dependent kinase inhibitor 1A (p21, Cip1)	M1775R*vs*WT	−0.3066647
	*CEBPA*	CCAAT/enhancer binding protein (C/EBP), alpha	M1775R*vs*WT Mut*vs*WT	−0.3728651
				−0.3190284
	*SMAD3*	SMAD family member 3	A1789T*vs*WT M1775R*vs*WT Mut*vs*WT	−0.2675322
				−0.4286813
				−0.3196246
	*CCND1*	cyclin D1	A1789T*vs*WT	0.3622112
	*PML*	promyelocytic leukemia	M1775R*vs*WT	−0.3045759
	*RUVBL1*	RuvB-like 1 (E. coli)	M1775R*vs*WT	−0.3028029
	*TXNIP*	thioredoxin interacting protein	A1789T*vs*WT	−0.3985633
	*RASSF1*	Ras association (RalGDS/AF-6) domain family member 1	A1789T*vs*WT	−0.2766158
Cell proliferation enhancement	*FOS*	FBJ murine osteosarcoma viral oncogene homolog	A1789T*vs*WT M1775R*vs*WT Mut*vs*WT	0.4515777
				0.4020256
				0.4365775
	*DUSP1*	dual specificity phosphatase 1	A1789T*vs*WT M1775R*vs*WT Mut*vs*WT	0.3844494
				0.7606655
				0.5060076
	*DUSP2*	dual specificity phosphatase 2	Mut*vs*WT	0.5408689
	*EDN1*	endothelin 1	M1775R*vs*WT Mut*vs*WT	0.4442705
				0.3212824
	*SKP1*	S-phase kinase-associated protein 1	A1789T*vs*WT	0.3353208
	*ZWILCH*	Zwilch, kinetochore associated, homolog (Drosophila)	A1789T*vs*WT	0.2508541
	*GPR56*	G protein-coupled receptor 56	A1789T*vs*WT M1775R*vs*WT Mut*vs*WT	−0.3453577
				−0.3310188
				−0.3407359
Apoptosis blocking	*NFKB 1*	nuclear factor of kappa light polypeptide gene enhancer in B-cells 1	M1775R*vs*WT	−0.2522979
	*TNFRSF10B*	tumor necrosis factor receptor superfamily, member 10b	M1775R*vs*WT	−0.247568
	*DYRK2*	dual-specificity tyrosine-(Y)-phosphorylation regulated kinase 2	M1775R*vs*WT	−0.282513
	*PLEKHF1*	pleckstrin homology domain containing, family F (with FYVE domain) member 1	Mut*vs*WT	−0.2374774

#### Cell cycle arrest impairment

CDKN1A, downregulated by M1775R, is a main effector of cell cycle arrest in response to DNA damage and a promoter of apoptosis [28]. Its expression is usually activated by BRCA1 [2].

Cell cycle can be also arrested by the cooperation of CDKN1A with CEBPA that was in turn downregulated by M1775R [29].

*CDKN1A* expression is normally activated also by SMAD3, a known transcription factor that acts as an effector of the TGF-beta pathway [30], downregulated in all the three comparisons. The overexpression of SMAD3 in a breast cancer cell line has been shown to cause cell cycle arrest [31], while in *SMAD3*−/− mammary epithelial cells, both TGF-beta-induced growth inhibition and apoptosis are lost [32].

SMAD3 also contributes to the 3-indole-induced G1 arrest in cancer cells [33] and its inhibition depends on CCND1-CDK4 (cyclin-dependent kinase 4) action in breast cancer cells overexpressing *CCND1*[34], which appeared upregulated by A1789T. The loss or reduction of BRCA1 expression, moreover, significantly reduces the TGF-beta induced activation of SMAD3 in breast cancer cells [35].

Four other genes linked to cell cycle control appeared downregulated, two, *PML* and *RUVBL1*, by M1775R and two, *TXNIP* and *RASSF1,* by A1789T*. PML* codifies for a phosphoprotein localized in nuclear bodies involved in recognition and/or processing of DNA breaks and able to arrest cell cycle in G1 by recruiting TP53 and MRE11A [36]; *RUVBL1* encodes a highly conserved ATP-dependent DNA helicase that plays a role in apoptosis and DNA repair [37]; *TXNIP* acts as a tumor suppressor, as its transfection induces cell-cycle arrest in G0/G1 and is downregulated in human tumors [38] and *RASSF1* is a tumor suppressor that blocks cell cycle progression by inhibiting CCND1 accumulation. It is epigenetically inactivated in many tumors, including breast cancer [39,40].

#### Cell proliferation enhancement

The transcription factor *FOS*, upregulated in all the three comparisons, is a well known protooncogene that positively regulates cell cycle progression [41] and is induced in human breast cancer cell cultures [25].

DUSP1, upregulated in all the three comparisons, and DUSP2, upregulated in Mut*vs*WT, belong to a subfamily of tyrosine phosphatases that regulate the activity of Mitogen-Activated Protein Kinases (MAPKs). MAPKs are key effectors for cell growth control and survival in physiological and pathological conditions, including cancer and DUSPs have been therefore proposed as potential targets for anticancer drugs [42]. DUSP1 inhibits apoptosis in human mammary epithelial and breast carcinoma cells [43] and its expression is upregulated in many breast cancers [44]. The overexpression of DUSP2 in ovarian cancers has been correlated with poor outcome [45].

EDN1, upregulated by M1775R and in Mut*vs*WT, is a vasoconstrictor that has also co-mitogenic activity, potentiating the growth factor effects. Altered EDN1 signalling is involved in carcinogenesis by modulating cell survival and promoting invasiveness [46].

SKP1, upregulated by A1789T, is a component of the SCF complex that mediates the ubiquitination of cell cycle proteins promoting cell cycle progression [47].

ZWILCH, upregulated by A1789T, is an essential component of the mitotic checkpoint that prevents cells from exiting mitosis prematurely [48].

*GPR56*, downregulated in all the three contrasts, is a G protein-coupled receptor involved in adhesion processes that participates in cytoskeletal signaling, cellular adhesion and tumor invasion. It is downregulated in melanoma cell lines, while its overexpression suppresses tumor growth and metastasis [49].

#### Apoptosis blocking

NFKB1, downregulated by M1775R, is a pleiotropic transcription factor involved in many biological processes like inflammation, immunity, differentiation, cell growth, tumorigenesis and apoptosis. Whether NFKB activation contributes or not to cancer is controversial [50], as it regulates the expression of both antiapoptotic [51] and proapoptotic genes [52,53].

Interestingly, *TNFRSF10B*, that was in turn downregulated by M1775R, is one of the proapoptotic genes upregulated by NFKB [53]. TNFRSF10B is one of the two apoptosis-activating receptors binding TNFSF10 (tumor necrosis factor (ligand) superfamily, member 10) [54] that, together with FADD (Fas(TNFRSF6)-Associated via Death Domain) forms a complex that leads to apoptosis through caspases activation [55].

DYRK2, downregulated by M1775R, is a protein kinase that regulates TP53 in inducing apoptosis in response to DNA damage [56] and PLEKHF1, downregulated in Mut*vs*WT, is a recently discovered lysosome-associated protein that activates apoptosis [57] by interacting with the TP53 transactivation domain [58].

### Genomic instability

An improper reaction to genotoxic stress causes genomic instability, leading to tumorigenesis. Deficiencies in DNA damage signaling and repair pathways are thus fundamental to the etiology of cancer [59].

Among the DEGs involved in genotoxic stress response, some were downregulated causing an increase in genomic instability, others were upregulated (Table 3). Many tumors, including BRCA1-deficient breast cancers, show an overexpression of genes linked to DNA repair that correlates with chemoresistance and poor prognosis [60,61]. Moreover, an increased nuclear staining of DNA repair proteins has been recently observed in tissue sections of breast cancers carrying the M1775R mutation, suggesting a new mechanism of tumorigenesis that involves an enhance of homologous recombination [62].

**Table 3:** Genes linked to genomic instability

**Biological Process**	**Gene Symbol**	**Gene Name**	**Contrast**	**log2 (Fold Change)**
DNA damage response and repair downregulation	*EEF1E1*	eukaryotic translation elongation factor 1 epsilon 1	A1789T*vs*WT	−0.4309041
	*SMC1A*	structural maintenance of chromosomes 1A	A1789T*vs*WT Mut*vs*WT	−0.2754507 −0.2640263
	*PPP1CC*	protein phosphatase 1, catalytic subunit, gamma isozyme	A1789T*vs*WT	−0.4286825
	*AHNAK*	AHNAK nucleoprotein	A1789T*vs*WT M1775R*vs*WT Mut*vs*WT	−0.3988113
				−0.3103867
				−0.3940570
	*SOD2*	superoxide dismutase 2, mitochondrial	M1775R*vs*WT Mut*vs*WT	−0.3376169
				−0.2502831
DNA damage response and repair upregulation	*MRE11A*	MRE11 meiotic recombination 11 homolog A (S. cerevisiae)	A1789T*vs*WT	0.3293561
	*TERF1*	telomeric repeat binding factor (NIMA-interacting) 1	Mut*vs*WT	0.2790907
	*OBFC2A*	oligonucleotide/oligosaccharide-binding fold containing 2A	M1775R*vs*WT	0.3666172
	*OBFC2B*	oligonucleotide/oligosaccharide-binding fold containing 2B	A1789T*vs*WT Mut*vs*WT	0.4070777
				0.3417360

#### DNA damage response and repair downregulation

EEF1E1, downregulated by A1789T, first discovered as associated with a macromolecular tRNA synthetase complex, is a key factor for ATM/ATR-mediated TP53 activation in response to DNA damage [63].

*SMC1A*, downregulated by A1789T and in Mut*vs*WT, encodes an evolutionarily conserved chromosomal protein, component of the cohesin complex [64]. SMC1A associates with BRCA1 and is phosphorylated in response to ionizing radiations in an ATM- and NBN-dependent manner [65].

PPP1CC, downregulated by A1789T, is the catalytic subunit of the gamma isoform of PP1 which is a component of a signaling complex, PPP1R1A/PPP1R15A/PP1 that positively regulates apoptosis in response to various stresses, including growth arrest and DNA damage [66].

*AHNAK*, downregulated in all the three contrasts, encodes a protein typically repressed in human neuroblastoma cell lines and in other types of tumors [67]. It firmly binds the LIG4-XRCC4 (ligase IV, DNA, ATP-dependent and X-ray repair complementing defective repair in Chinese hamster cells 4) complex on DNA stimulating its double-stranded ligation activity [68].

SOD2, downregulated by M1775R and in Mut*vs*WT, is a member of the iron/manganese superoxide dismutase family that acts as a free radical scavenger. It is a candidate tumor suppressor gene as the loss of heterozigosity of its region on chromosome 6 has been found in about 40% of human malignant melanomas [69] and the deletion of chromosome 6 long arm has been identified in SV40 transformed human fibroblasts [70]. In addition, SOD2 overexpression suppresses the tumorigenicity of breast cancer cells [71].

#### DNA damage response and repair upregulation

*MRE11A*, upregulated by A1789T, encodes a component of BASC (Brca1 Associated genome Surveillance Complex), which specifically promotes non-homologous end-joining [72,73]. Interestingly, the A1789T variant altered the non-homologous end-joining activity in a functional assay [11].

TERF1, upregulated in Mut*vs*WT, is a telomere-associated protein, member of the telomere nucleoprotein complex that interacts with various polypeptides, like the MRN complex [74].

*OBFC2A*, upregulated by M1775R, and *OBFC2B*, upregulated by A1789T and in Mut*vs*WT, encode single-stranded DNA-binding proteins essential for DNA replication, recombination and damage detection and repair. OBFC2B, in particular, as an early participant in DNA damage response, is critical for genomic stability [75].

## Conclusions

As we first observed in yeast cells [11], also in human cells the *BRCA1* variants M1775R and A1789T affect the expression of many genes critical for cell proliferation and genome integrity maintenance. Our results represent the first molecular confirmation of the pathogenetic role of M1775R. In fact, although more than an evidence exists on the pathogenetic role of this BRCA1 variant, the effect of this mutation on human cell transcriptome has never been investigated before.

Concerning the A1789T variant, it has been studied only by our group. On the basis of experiments in yeast, we previously suggested for this mutation a causative role in breast cancer onset and development similar to that of M1775R. The present work gives further support to this hypothesis.

## Competing interests

The authors declare that they have no competing interests.

## Authors’ contributions

CI contributed to conceive the study, carried out the RT-qPCR experiments, performed the biological interpretation of microarray data and drafted the manuscript. EM conceived the experimental design, performed the statistical analysis and contributed to draft the manuscript. VM carried out the microarray experiments and contributed to draft the manuscript. CG carried out the western blot experiments and contributed to the biological interpretation of microarray data. LG performed the cell transfection. MAC contributed to conceive the study and to the writing up of the manuscript. SP conceived the study, supervised the experiments, contributed to the interpretation of the results and to the writing up of the manuscript. All authors read and approved the final version of the manuscript.

## Pre-publication history

The pre-publication history for this paper can be accessed here:

http://www.biomedcentral.com/1471-2407/12/207/prepub

## Supplementary Material

Additional file 1 **Microarray results of Mut*vs*WT-contrast.** The four tabs contain the DEGs, the pathway analysis results and the mapped genes by Pathway-Express and the ontological analysis results by Onto-Express, respectively. Click here for file

Additional file 2 **Microarray results of M1775R*vs*WT-contrast.** The four tabs contain the DEGs, the pathway analysis results and the mapped genes by Pathway-Express and the ontological analysis results by Onto-Express, respectively. Click here for file

Additional file 3 **Microarray results of A1789T*vs*WT-contrast.** The four tabs contain the DEGs, the pathway analysis results and the mapped genes by Pathway-Express and the ontological analysis results by Onto-Express, respectively. Click here for file

Additional file 4 Intersections among the three lists of DEGs. Click here for file

Additional file 5 Intersections among the three lists of pathways. Click here for file

Additional file 6 Intersections among the three lists of DEGs and the list of genes related to "Cell Proliferation" biological term by Coremine. Click here for file

Additional file 7 Intersections among the three lists of DEGs and the list of genes related to "DNA damage and repair" biological term by Coremine. Click here for file
